# Stage I endometrial adenocarcinoma with a vaginal nodule—the importance of differential diagnosis

**DOI:** 10.1097/j.pbj.0000000000000168

**Published:** 2022-12-01

**Authors:** Isabel Rodrigues, André Laranja, Luísa Carvalho, Lurdes Salgado

**Affiliations:** a External Radiotherapy Department, Institute Português de Oncologia do Porto Francisco Gentil, EPE, Portugal,; b Brachytherapy Department, Instituto Português de Oncologia do Porto Francisco Gentil, EPE, Portugal.

**Keywords:** endometrial cancer, brachytherapy, differential diagnosis, leiomyoma, neoplasm, metastasis

To the Editor

Endometrial cancer is a common malignancy of the female reproductive system. Most are early-stage but can spread locally and metastize (also to the vaginal wall). We report 2 clinical cases of post-menopausal women with early-stage endometrial cancer, after hysterectomy, who were offered adjuvant brachytherapy as monotherapy according to pathological stage and histology. On gynecological examination before brachytherapy, a vaginal nodule was identified in both patients, clinically compatible with a metastatic lesion. However, a biopsy was requested and histology classified both lesions as vaginal leiomyomas. The patients then proceeded with their initial strategy.

It is therefore imperative to obtain histological confirmation of any new finding that may restage and alter the planned adjuvant treatment, to better adjust treatment to the correct stage, predict prognosis, and avoid iatrogeny. In this article, we highlight the importance of a thorough differential diagnosis and biopsy confirmation of any vaginal lesion that may change the adjuvant management of an early-stage endometrial tumor, and review published literature on vaginal leiomyomas.

## Introduction

Endometrial cancer is a common malignancy of the female reproductive system, accounting for around 380,000 new cases worldwide each year.^1^ Although the vast majority present in stage I,^2^ they can spread locally and metastize as well, most commonly to lung, liver, or bone, and less frequently to the vaginal wall by lymphatic/hematogenous spread or implantation after surgery (in nearly 10% stage I endometrial carcinomas).^2^ According to several international guidelines, these stage I tumors are treated with surgery (total hysterectomy and bilateral salpingoophorectomy, with or without surgical nodal staging according to stage), followed by adjuvant vaginal brachytherapy in monotherapy or pelvic radiotherapy with or without chemotherapy, according to stage, grade, lymph vascular space invasion, and whether nodal staging was performed (IAG1-3/IBG1-2 tumors with lymph vascular space invasion without nodal staging and IBG3 tumors may be offered adjuvant pelvic radiotherapy with or without chemotherapy). Low-risk tumors (IAG1-2 without lymph vascular space invasion) do not undergo adjuvant treatment.^3^

Since vaginal metastasis would upstage the disease, changing the adjuvant strategy (that would also then imply chemotherapy and external radiotherapy), histological confirmation is crucial when a suspected lesion is identified. Here, we present 2 clinical cases referred to our institution for adjuvant brachytherapy in monotherapy that presented with a vaginal nodule in the gynecological examination. The purpose of this paper is to highlight the importance of a thorough differential diagnosis, to better adapt the adjuvant strategy.

## Case descriptions

### Case 1

We report the case of a 71-year-old post-menopausal woman with a 3-month history of metrorrhagia. She had no relevant medical history. She underwent a transvaginal ultrasound that showed endometrial thickening and was referred to a gynecologist in February 2019. Hysteroscopy with biopsy established the diagnosis of endometrioid adenocarcinoma grade 3, and metastatic involvement was excluded via computed tomography. Surgery was performed in March 2019 (hysterectomy, bilateral salpingoophorectomy, pelvic and lumboaortic lymphadenectomy, and peritoneal washing). Pathology described a 2cm polypoid lesion (corresponding to the endometrioid carcinoma) on the fundus, invading less than halfway through the myometrium, with negative margins, without tumor on the cervix or adnexa. Three uterine subserosal leiomyomas up to 2 cm were also present. No lymph nodes were involved (5 were excised from the right pelvis, 4 from the left pelvis, and 8 from the lumboaortic region) and the peritoneal washing product was negative for malignancy. The tumor was staged as pIAG3-FIGO (intermediate-high risk according to the Portuguese Consensus on Gynecologic Oncology).^3^ The patient was offered adjuvant vaginal brachytherapy and referenced to our Institution in May 2019. On the first gynecological exam at our department, a 1 cm vaginal nodule was identified on the superior third of the posterior vaginal wall, with a granular and friable appearance. It was palpable via rectal examination and suspected to be metastatic. An excisional biopsy was performed, and analysis described a vaginal leiomyoma (common pattern), with no evidence of malignancy. Since the hypothesis of secondary vaginal involvement was excluded, the patient underwent the planned adjuvant strategy (3 weekly fractions of 7Gy high dose rate endovaginal brachytherapy with Iridium-192, to a vaginal extension of 3 cm and prescribed to a 5 mm depth of the vaginal mucosa, which she completed at the beginning of July 2019). She remains in follow-up, with no recurrence.

### Case 2

We refer to the case of a 76-year-old post-menopausal woman, with no relevant medical history. She was referred to a gynecologist in March 2019 with an 18-month history of vaginal bleeding, after an ultrasound that identified a 9 mm endometrial thickening and a subserous 4 cm uterine myoma. Hysteroscopy and curettage were performed, and pathology reported an endometrioid adenocarcinoma grade 1. Computed tomography excluded the presence of secondary involvement and the patient underwent surgery in September 2019 (hysterectomy, bilateral salpingoophorectomy, pelvic and lumboaortic lymphadenectomy, and peritoneal washing). Pathology described a 5 cm infiltrative lesion in the uterine cavity (confirming the diagnosis of endometrioid carcinoma and reclassifying it as grade 2), invading the outer half of the myometrium (1 mm from the serosa), without lymph vascular permeation, cervical, or adnexal invasion. A total of 8 right pelvic, 5 left pelvic, and 6 lumboaortic lymph nodes were isolated, without malignant involvement. Peritoneal fluid showed no evidence of malignancy. The patient was then staged as pIBG2-FIGO (intermediate risk).^3^ She was referred to our institution after being offered adjuvant brachytherapy. She was examined in our department in October 2019, and a 1 cm nodule on the anterior left wall of the vagina was identified, which was hard and not distinguishable on inspection, suspected of being malignant. An excisional biopsy was requested and a partial colpectomy was performed in December 2019, excluding the presence of malignant cells, and characterizing the nodule as a common pattern vaginal leiomyoma. The patient then proceeded to the planned adjuvant strategy and completed vaginal brachytherapy in late January 2020 (after 3 weekly fractions of 7Gy high dose rate endovaginal brachytherapy with Iridium-192, to a vaginal extension of 3 cm, prescribed to 5 mm beyond the surface of the vaginal mucosa). She has no evidence of recurrence to date.

## Discussion

Vaginal leiomyomas are rare benign entities, with around 300 reported cases.^4^ Most are located on the anterior vaginal wall and are generally small and asymptomatic,^5^ manifesting only after increasing in size, causing dyspareunia, bleeding, purulent discharge, urinary obstruction, or/and pain.^6,7^ These are estrogen-related tumors, most common in caucasian women in the 35–50 years age group.^4^ Treatment is surgery with free margins and, when a large mass is present, may entail abdominal and perineal approaches.^7^ Surgery should be performed as soon as the lesion is detected since there is a risk of sarcomatous transformation.^5^ When suspected, and depending on the size, computed tomography or magnetic resonance imaging is generally requested to assess the extension of the mass, but the final diagnosis is generally only reached after histological examination, which is considered the gold standard.^4^

In these 2 clinical cases, vaginal leiomyomas presented as incidental findings on physical examination before adjuvant vaginal brachytherapy for endometrial adenocarcinoma (not having been described in previous examinations). They presented similarly to metastatic lesions, and therefore excisional biopsies were requested. Isolated vaginal metastasis/recurrence is more commonly identified in stage I or II diseases in patients who did not receive adjuvant radiotherapy, more commonly in the apex, followed by the middle vaginal canal, and more rarely in the distal vagina and suburethral area. It correlates with involvement of the cervix, which these patients did not present with.^8^

Adjuvant radiotherapy (external beam radiotherapy or brachytherapy) is recommended after surgery for most stages of endometrial carcinoma, having been associated with a reduction in a locoregional relapse in several studies, such as GOG-99 and PORTEC-1.^9,10^ According to PORTEC-2, adjuvant brachytherapy is the preferred approach in intermediate and intermediate-high risk patients, with similar vaginal relapse rates and overall survival comparing with adjuvant external beam radiotherapy while exhibiting a more favorable toxicity profile.^11^ According to their stage and risk group, both patients were hence offered adjuvant brachytherapy, with the widely used PORTEC-2 regimen, having received 3 fractions of 7Gy.^11^

Upon the identification of the vaginal nodules, the goal of the biopsies was to aid in decision-making, since the presence of metastatic vaginal lesions would upstage the disease to stage IIIB, constituting an indication for chemotherapy and external pelvic radiotherapy. When comparing with a brachytherapy-only adjuvant strategy, these treatments would induce several early and late side effects with a significant impact in the patients’ quality of life, both on short (the most common being alopecia, diarrhea, cytopenia, pain, neuropathy, nausea, and fatigue) and long term (with reported grade 2 alopecia in 19%, hematological effects in 16%, neuropathy in 13%, pain in 9%, gastrointestinal effects in 6%, and G3–4 effects in 8% with modern techniques).^12^ A decision to escalate adjuvant treatment would then have to be based on solid clinical evidence.

These 2 clinical cases illustrate the importance of a thorough differential diagnosis between a vaginal metastasis and other malignant or benign lesions, such as vaginal leiomyomas. These rare entities presented as small nodules in both patients, and the final diagnosis was established by histopathology. This highlights the importance of thoroughly documenting benign lesions found on physical examination, as well as obtaining a biopsy specimen to avoid unnecessary and inadequate changes in the therapeutic plan.
